# Tic Detection in Tourette Syndrome Patients Based on Unsupervised Visual Feature Learning

**DOI:** 10.1155/2021/5531186

**Published:** 2021-06-07

**Authors:** Junya Wu, Tianshu Zhou, Yufan Guo, Yu Tian, Yuting Lou, Hua Ru, Jianhua Feng, Jingsong Li

**Affiliations:** ^1^Engineering Research Center of EMR and Intelligent Expert System, Ministry of Education, Key Laboratory for Biomedical Engineering of Ministry of Education, College of Biomedical Engineering and Instrument Science, Zhejiang University, Hangzhou 310027, China; ^2^Research Center for Healthcare Data Science, Zhejiang Lab, Hangzhou 311100, China; ^3^Department of Pediatrics, The Second Affiliated Hospital of Zhejiang University School of Medicine, No. 88 Jiefang Road, Hangzhou 310009, China

## Abstract

A clinical diagnosis of tic disorder involves several complex processes, among which observation and evaluation of patient behavior usually require considerable time and effective cooperation between the doctor and the patient. The existing assessment scale has been simplified into qualitative and quantitative assessments of movements and sound twitches over a certain period, but it must still be completed manually. Therefore, we attempt to find an automatic method for detecting tic movement to assist in diagnosis and evaluation. Based on real clinical data, we propose a deep learning architecture that combines both unsupervised and supervised learning methods and learns features from videos for tic motion detection. The model is trained using leave-one-subject-out cross-validation for both binary and multiclass classification tasks. For these tasks, the model reaches average recognition precisions of 86.33% and 86.26% and recalls of 77.07% and 78.78%, respectively. The visualization of features learned from the unsupervised stage indicates the distinguishability of the two types of tics and the nontic. Further evaluation results suggest its potential clinical application for auxiliary diagnoses and evaluations of treatment effects.

## 1. Introduction

Tourette syndrome (TS) is a childhood-onset neurodevelopmental disorder characterized by the presence of fluctuating motor and vocal tics [1]. The core diagnostic features are both multiple motor and one or more phonic tics lasting more than one year. Typically, the same tic occurs at short-term periodicity with short intervals [2]. The simple tic forms are eye blinking, mouth twitching, head jerking, etc. Multiple studies published since 2000 have consistently demonstrated that the prevalence of TS is much higher than previously thought [3]. As the understanding of this disease deepens, the number of children diagnosed with tic disorder has gradually increased, but most cases do not receive timely clinical attention in the early stages of the disease. Furthermore, approximately 20% of persons with TS are unaware that they have tics [4]. The clinical diagnosis of TS involves complex processes that require considerable time and effective cooperation between the doctor and the patient, especially observation and evaluation of the patient's tic behaviors. A number of instruments for tics and associated phenomena have been developed to assess tic severity [5] and differ in construct, comprehensiveness, and ease of administration.

Recently, artificial intelligence and machine learning have been widely applied in the medical field. In particular, the development of video-based human motion behavior analysis technology has advanced various types of medical diagnoses, such as Parkinson's disease [6], seizure disorders [7], spinal muscular atrophy [8], and discomfort detection in premature infants [9]. In part, noncontact video-based analysis has attracted great attention due to the increasing availability of camera monitoring systems. To identify tic disorders, patients' tic movements can be detected and analyzed from video tapes that show the patient's face, head, or body and rated according to the Yale Global Tic Severity Scale (YGTSS) [10] or the Modified Rush Video-based Rating Scale (MRVRS) [11]. These ratings can then be used to assist a clinical doctor in evaluating the patient's symptoms and severity.

Tic movements can be distributed throughout the body. Rickard's research [12] showed that patients' twitches usually start in the facial area and that eye twitches are the most frequent. Chappell et al. [13] showed that, in addition to a severity scale, the severity of Tourette syndrome can also be determined by recording the patient's tics with video for more than ten minutes. Moreover, monitoring and recording patients in their natural states instead of facing a clinician effectively avoids interference in diagnosis and evaluation caused by the patient actively controlling their tics. Therefore, we aim to develop a method to automatically detect tics to help clinicians or parents spot and assess tic signs.

In recent decades, many studies have focused on the pathology, genetics, and clinical treatment of TS [14–16], but only a few studies have been published regarding the automatic detection of TS-related motor disturbances. Jonathan et al. [17] studied two patients with TS using deep brain stimulation (DBS) during tics and found that low-frequency (1-10 Hz) centromedian (CM) thalamic activity and beta frequency motor cortex (M1) activity were tic features and that long complex tics are concurrent with a highly detectable thalamocortical signature. Bernabei et al. [4] used a wearable device attached to the patient's trunk with an embedded triaxial accelerometer to monitor tic events. This approach achieved a sensitivity of 80.8%, a specificity of 75.8%, and an accuracy of 80.5%. However, the implementation process of this method is quite complicated, which poses a major challenge and requires extensive cooperation between doctors and patients. Recently, Barua et al. [18] proposed a deep learning approach for detecting tic disorders using wireless channel information and achieved an accuracy above 97%. The data used in the task were simulated using healthy human subjects. However, in a real clinical situation, acquiring such data would be a considerably more complicated task. Regarding methodological aspects, action detection methods have made numerous advancements in video comprehension, such as the two-stream network [19–21], 3D ConvNet [22–24], and temporal enhancement-and-interaction network (TEINET) [25], whereas these deep learning networks require large amounts of labeled data, which carries the high costs and slow procedures associated with manual labeling. Data labeling is often costly and time consuming: an example is the popular ImageNet dataset [26]. However, in real-world situations, large amounts of readily accessible unlabeled data exist; therefore, unsupervised learning has attracted increasing attention from researchers.

From these perspectives, we instead adapt a two-stage architecture by first training an unsupervised feature extraction model to make full use of the more easily acquired unlabeled data and then applying a comparatively simple network attached to the former trained model for the classification tasks. Visualizing the feature representation of the labeled data shows the correspondence with the tic parts, indicating that the unsupervised model learned the valid feature representation. This approach results in the following contributions: (1) we employ a deep learning scheme by a convolutional-neural-network- (CNN-) based model to learn feature representation from abundant unlabeled video data, (2) we apply a long short-term memory neural network (LSTM) to classify the feature sequences of video clips, and (3) an automated video-based system for detecting tic movements in TS patients is devised.

## 2. Materials and Methods

**Figure 1:**
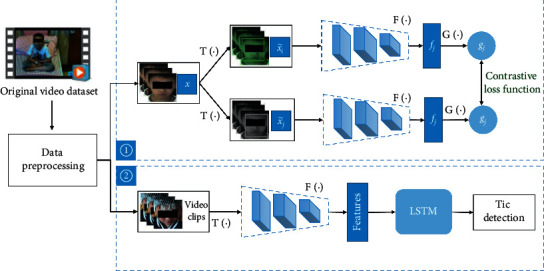
The architecture of the proposed method. (1) Stage 1: extracting representative visual features. (2) Stage 2: training an LSTM using visual features.

To solve the problem of insufficient labeled data but enough monitoring video data, we propose a two-stage framework that combines unsupervised and supervised learning, as shown in Figure 1. In the first stage, we adopt a contrastive learning network that learns from unlabeled video data by extracting features by maximizing mutual information. The core idea behind this is to maximize the mutual information between the two nonoverlapping patch inputs. In the second stage, we design an end-to-end architecture based on an LSTM network connected to the feature extraction module in the first stage that learns to classify tic movements from video data labeled by doctors. We use a combination of supervised and unsupervised learning to design and build an end-to-end tic detection model.

### 2.1. Subjects

Sixty-eight patients (4–13 years old) diagnosed with TS by two experienced specialists were employed in this study. All participants were inpatients under normal treatment recruited from the Second Affiliated Hospital of Zhejiang University School of Medicine between May and September 2019. This study was approved by the ethics committee of the Second Affiliated Hospital of Zhejiang University School of Medicine (YAN2019-148). All participants provided written informed consent with the agreement to participate in the study.

### 2.2. Data Acquisition and Preprocessing

The TS dataset was sourced from the Department of Pediatrics at the Second Affiliated Hospital of Zhejiang University School of Medicine and was collected using EEG video acquisition equipment installed in the pediatric ward. The video data were recorded in two situations: (i) the patient was asked to sit on a chair in front of the camera and (ii) before or after EEG recording, the patient was asked to agree to video recording. The two situations arise because the data were collected in different periods: (i) represents data collected during the preproject preparation phase, whereas (ii) is a part of the routine during subsequent EEG video recording. In both situations, every patient was informed in advance of the recording period and asked to face the camera as much as possible during recording, but no mandatory measures were imposed; the patients could move freely, which may result in useless frames. The patients' parents provided informed permission for the collection procedure and research use of the video recordings.

Due to different camera devices, the original video frame rate includes video acquired at both 30 and 25 fps. The duration of all videos ranges from 5 to 15 minutes, and all the videos have a resolution of 1920 × 1080. The length of the videos is listed in Table 1, and the distribution of the durations is presented in Figure S1. In this TS dataset, 13 cases were annotated by two specialists, who labeled the starting and ending timestamps of a specific tic event, such as an eye tic or a mouth tic. They performed manual annotation frame by frame through the video annotation software VoTT (https://github.com/microsoft/VoTT), which then generated annotated JavaScript Object Notation (JSON) files for postprocessing to extract annotations. The annotation work was independently performed by two clinicians and verified by the third expert, and they performed an extra check if there were disagreements until they reached a consensus. Finally, we cut and categorized the videos based on the timestamped annotations to form the TS research dataset, which can be supplemented at any time. We also segmented the video segments between two labeled tic events in the original video, which can be used as normal recordings and act as negative samples.

**Figure 2:**
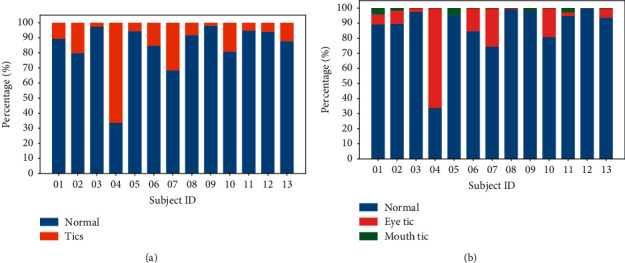
Category proportion of normalized video clips of patients in (a) the multiclass classification task and (b) the binary classification task. The ordinate indicates the patient number in the labeled TS dataset.

There are more than five types of tics involving different muscle groups in the labeled data. The most common tics are eye tic and mouth tic. Not only in this research dataset but also most in clinical practice, these two tic types are widespread from a specialist's perspective [12]. Therefore, we defined two classification tasks. (1) We chose these two tic types and normal recordings to define a multiclass classification task. (2) We also configured a binary classification task for the tic and normal datasets—that is, all the tic video clips form the positive samples, while the normal video clips form the negative samples.  Figure 2 shows the category proportion of every patient in the labeled dataset, defined in these two tasks.

**Figure 3:**
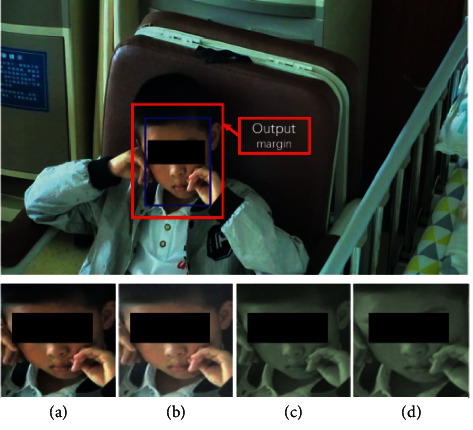
Region of interest in data preprocessing. (a) is the output of the MTCNN, and (b), (c), and (d) are the random data augmentation methods applied.

The proposed method is composed of two stages, and there are slight differences in data preprocessing for the two stages. The common operation is to obtain the region of interest (ROI), which is defined as the area centered on the patient's face. This ensures that the models will focus on features related to patients' tic behaviors rather than on other family members or physicians visible in the videos. Identifying the ROI also reduces interference from different camera angles and from patient movements since they are free to move out of the camera view. This procedure uses a neural-network-driven face detection method. We use the multitask cascaded CNN (MTCNN) [27] architecture to detect the patient's face and obtain the ROI and use the pretrained weights from Face2 [28]. To avoid the regional deviations caused by free patient motion and obtain more features in the face area, we extract the ROI area by expanding the width of the face bounding box by 20%.  Figure 3 illustrates the ROI output margin. We also conduct data augmentation during preprocessing, an approach that has been widely used in both supervised and unsupervised learning [29, 30]. The effectiveness of simple data augmentation methods for contrastive learning tasks was verified by [31]. Similarly, after obtaining the ROI area, combined data augmentation methods are adopted including random cropping, random noise, and random color distortion.

The difference between the two main stages during data preprocessing is that the first stage uses a relatively large number of frames from an unlabeled video dataset. As well known, the information between continuous video frames is usually highly redundant, which can cause overfitting during training. Thus, we perform a 3-fold downsampling procedure, which extracts the first frame for every three consecutive frames on the original videos. In the second supervised training stage, we first cut the original continuous video data into video segments and divide them into motion tic categories based on annotations. Then, we cut each video segment into 1 s video clips using a no overlapping sliding window. These clips form the input objects for Stage 2. We then performed the same ROI extraction procedure as for Stage 1 but without video frame downsampling because the frame data are randomly extracted from every second of input, which has an effect similar to downsampling.

### 2.3. Stage 1: Extracting Representative Visual Features

The dataset used in this work consists of a small amount of labeled data (*n* = 13) and a relatively large amount of unlabeled data (*n* = 55). Apparently, the labeled data we have are insufficient to train a deep learning model. To explore the value of the unlabeled data, we adopt a contrastive learning framework similar to SimCLR [31] in Stage 1 to extract representative visual features among the TS patient groups. Specifically, a randomly selected minibatch S of N examples is transformed into a minibatch pair S′ consisting of 2N examples after applying a combination of a set of data augmentation methods. Then, S′ is input to the defined contrastive prediction task. For every minibatch pair S′, each pair (i, j) of augmented examples S′ (i, j) is treated as a positive example (*n* = 2), while the others (*n* = 2(N-1)) are treated as negative examples. Then the similaritysim(*i*, *j*) of the pair *S*′(*i*, *j*) is defined as follows:

(1) $$\begin{array}{c} \text{sim}\left(i,j\right)=\frac{S_{i}^{\prime T}S_{j}^{\prime }}{\left(\left\|S_{i}^{\prime }\right\|\left\|S_{j}^{\prime }\right\|\right)}, \end{array}$$

and the loss function of the pair loss(*i*, *j*) is defined as

(2) $$\begin{array}{c} \text{loss}\left(i,j\right)=-\log\frac{\exp\left(\text{sim}\left(i,j\right)/\tau \right)}{\sum_{k=1,k\neq i}^{2N}\exp\left(\text{sim}\left(i,k\right)/\tau \right)}, \end{array}$$

where *τ* denotes a temperature parameter, as in [32]. For each pair in every minibatch, the total loss is computed as follows:

(3) $$\begin{array}{c} L=\frac{1}{2N}\sum_{k=1}^{N}\left[\text{loss}\left(2k-1,2k\right)+\text{loss}\left(2k,2k-1\right)\right]. \end{array}$$

As shown in Figure 1, we use ResNet [33] as the neural network encoder (F) to extract the visual features after data augmentation, and we use an MLP network (*G*) to map the output feature *f* to the space where contrastive loss is applied. The contrastive prediction task involves finding the other example *j* in examples *S*′(*i* ≠ *j*)(*n*=2*N* − 1) for example *i* in each pair. In addition, we impose a restriction that every minibatch input must be a set of continuous frames randomly selected from the video frames of a single subject. This restriction eliminates the possibility of finding existing macrofeatures between different faces during training and helps the model focus on the microfeatures of tics.

### 2.4. Stage 2: Training the LSTM through Visual Features

In Stage 2, we design a supervised learning framework based on the formerly trained neural network encoder (F). The LSTM network consists of a layer of LSTM with dropout and a fully connected layer with rectified linear unit (ReLU) activation. Specifically, we take a one-second-long preprocessed video clip as the input of this stage and randomly select *k* frames (*k* < 25) to feed to F, which generates visual feature vectors. Every visual feature vector came from one frame of the input video clip and corresponded to one neuron of the LSTM layer. These visual feature vector sequences are then fed to the LSTM network to learn their temporal features and deeper spatial features to accomplish the classification task.

To alleviate the problem of imbalanced categories in our labeled data, we use the focal loss [34] *L*_*f*_, which is defined as follows:

(4) $$\begin{array}{c} L_{f}=-\alpha_{t}{\left(1-p_{t}\right)}^{\gamma }\log\left(p_{t}\right),\;p_{t}=\left\{\begin{array}{cc} p, & \text{if }y=1,\\ 1-p, & \text{otherwise,} \end{array}\right. \end{array}$$

where *y* denotes a tic label, *p* is the output prediction of the LSTM network, and *α* and *γ* are network hyperparameters.

## 3. Evaluation and Results

### 3.1. Experimental Setup

Chen et al. [31] showed that the simple operation of expanding the batch data volume can replace the more complex memory library training model [35, 36]. In this work, the two stages are trained separately. In Stage 1, we set the batch size to 512 and limited the single-input data to a randomly chosen person's continuous data due to the memory limitation of the training platform. For the neural network encoder, we used a modified ResNet18 model, with an input dimension of 112 × 112 × 3 and an output dimension of 512. The following MLP network consists of two layers: the first layer has 512 neurons and the second layer has 256 neurons. All the preprocessed unlabeled video datasets were randomly split into a training set (70%) and a validation set (30%) at the patient level. During training, we used the Adam optimizer [37] with an initial learning rate of 3 × 10^−4^ adjusted by setting the cosine annealing learning rate (LR) and a weight decay of 10-6. Considering the limitation of our dataset, we used the pretrained built-in weights of PyTorch [38]. The training procedure is stopped when the loss of validation set has no more drops within 10 epochs. Then the ResNet model is reused in Stage 2 to extract feature representations.

In Stage 2, each video clip generates a feature vector of clip-length × 512 through F. Here, clip-length is set as 16, which means that there are 16 frames randomly sampled from each video clip, matching the time-step setting in the LSTM network. The input size of the LSTM is 512 with a drop rate of 0.8; the output size is 128; and the size of the fully connected layer is changed to match the number of classes in each classification task. Considering the limited amounts of labeled data in the study that can easily cause overfitting during training and validation, we adopted the leave-one-subject-out cross-validation scheme in Stage 2, which allows us to evaluate the differences between individual patients. The setting for the overall analysis of a single patient is in line with the real clinical scenario, which is beneficial for the subsequent comprehensive analysis. We assess the effectiveness of our proposed method by calculating the accuracy, precision, recall, F1-score, area under the receiver operating characteristic (ROC) curve (AUC_ROC), area under the precision-recall curve (AUC_PR), and a confusion matrix for each subject evaluation. In the two different classification tasks, we consider different cutoff conditions during the training procedure by observing the following indicators from the validation evaluation: (1) accuracy and (2) the F1-score of the tic category. In addition to the data used for experimental modeling, we also collected individual test video data beyond those used for training verification to verify the universality of the method.

The next subsections report the details of the results and provide discussions. Unfortunately, to the best of our knowledge, no public TS dataset for tic detection exists, which makes it difficult to compare the results of our method with other works. Instead, we applied another two kinds of supervised ConvNet architectures, convolutional 3D (C3D) [22], and temporal segment network (TSN) [39], for comparison.

### 3.2. Classification Tasks

C3D [22] is a simple yet effective model that uses 3D convolution kernels for spatiotemporal feature learning, and TSN [39] combines a sparse temporal sampling strategy and video-level supervision. They both achieved good performances for action recognition in videos when given limited training samples. As shown in Table 2, compared with the former two approaches C3D [22] and TSN [39], our method with the watch-accuracy strategy achieves the best performances, with an average accuracy of 94.87%, precision of 86.26%, and both recall and F1-scores of approximately 80%. These results illustrate the effectiveness of our proposed method for tic recognition on the multiclass classification task.

**Figure 4:**
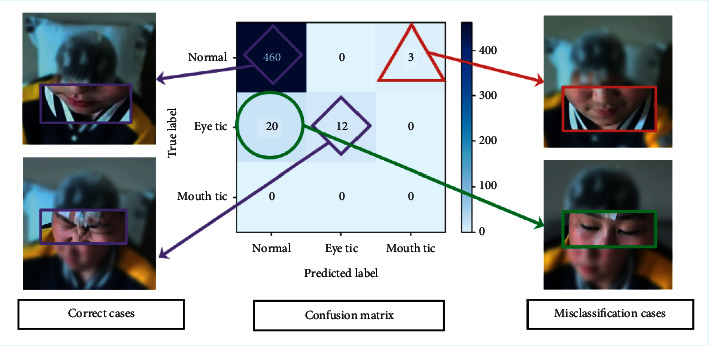
Evaluation result of one subject. The confusion matrix is shown in the middle; the correct detection cases from the multiclass classification task are shown on the left; and the misclassification cases are shown on the right. For the sake of patient privacy, the images used in the cases were blurred.

Using the classification results for an individual subject, we further examine the misclassified items. Taking Case 1 as an example, as shown in Figure 4, after checking the original data, we found that (a) in the false positive result where the label is normal but the prediction is mouth twitching, the mouth of the patient in this video clip does indeed twitch in the corners during a smile, indicating that the classification model has learned the features of the motion but cannot precisely differentiate between a mouth-twitching motion and a mouth-smiling motion when both are subtle; thus, it misclassifies the action. (b) In false negatives where the labels are eye tics while the prediction is normal, the patient in this video clip is indeed blinking, but it is difficult for ordinary people and for the model to determine whether the blink is normal or a twitch blink. This may be due to model misunderstanding, but a small possibility of labeling error also exists. These two situations reflect either possible misclassifications or misdiagnosis in the real situation; however, it should be noted that the identification unit in this study is at the level of a video clip, whereas the identification unit in the clinic is the complete subject over time. A few misidentifications from the clip may not be completely reflected at the level of subject recognition. Therefore, quantitative methods and full quantification of the video data for the standard duration at the subject level will be considered in future research. This will allow individual subject evaluations to be made and improve the model's application prospects for clinical auxiliary diagnosis.

The evaluation results of the binary classification task are shown in Table 3. Compared with the multiclass classification task, the indicator results are slightly lower in Table 2, and its watch-F1 strategy performs better. After comparing the datasets of the two tasks, we find that the multiclass data use two types of tics with more discriminative characteristics, which provides a data quality advantage. In contrast, the positive samples in the binary classification task cover all the tic categories that appear in the TS dataset. Despite these shortcomings, our model still achieves good recognition performance and will offer substantial clinical value after further optimization.

### 3.3. Further Evaluation

**Figure 5:**
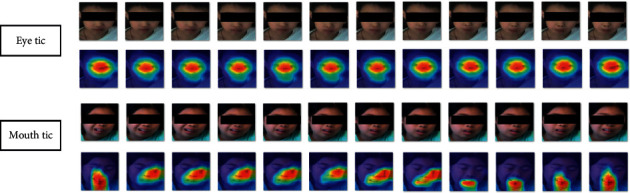
Visualization of two tic video clips of representation learning in Stage 1. The first row shows the original video clip frames; the second row shows the corresponding CAM image.

To verify the visual representation performance of the unsupervised model in Stage 1, we visualized the final layer features of the neural network encoder using the gradient-weighted class activation mapping (Grad-CAM) [40] method. As shown in Figure 5, the feature attention areas are shown as heatmap colors, and the attention areas are consistent with the corresponding tic positions, indicating that the unsupervised model has effectively learned the visual features used in the follow-up training.

To verify the validity of the proposed method and the possibility of subsequent integration with scales such as MRVRS [11], we compared the differences between the model's output and the clinician's result. This comparison test was based on the labeled dataset using the leave-one-subject-out test. We used two items based on the MRVRS and modified it within our data condition and one item for time comparison. The number of tic areas came from the annotations performed by clinicians and the tic categories of the model output. The tic frequency was calculated as the number of tic signs divided by the total length of the video used for every patient. Then, a *t*-test was performed on each of the items. The time for evaluation for clinicians was recorded between the start and the end for each video evaluation, and for our model it was calculated as the sum of the time taken for the whole process of our architecture, including preprocessing, model calculation, and postprocessing, among which preprocessing is the most time-consuming process. The subitem clinician review refers to the time taken for the clinician's checking process on the results of our models, which is divided into two categories: <5 min (0−5 min) and <10 min (5−10 min). The results are listed as Table 4. The *p* values of the two scale-related items are greater than 0.05, which shows no significant difference between the two groups of results. The *p* value of the time comparison is less than 0.0001, showing that our model can save considerable time on tic detection, especially for videos with frequent tic events, which indicates great potential for clinical application.

Further evaluation for independent testing was additionally conducted on new data to identify tic events, including 1 new patient video and 4 non-TS patient videos. The binary classification task was adopted for this experiment. In the non-TS patient video testing, as shown in Table 5, the recognition accuracy for every non-TS patient was above 90%; the highest accuracy exceeded 98%. As discussed in the preceding subsection, the evaluation scores are computed at the video-clip level. If that were to be upgraded to the subject level in a clinical application, this level of individual evaluation would be acceptable. For the patient data, we must take recall into account; that is, the tic detection accuracy reaches 72.69%. Although we have only one testing video for this initial study, while these results lack statistical significance, they can still indicate that this approach has optimistic application prospects.

## 4. Discussion

Tourette syndrome is a highly individualized neurological disease whose expression changes over time. In the process of long-term observation, diagnostic evaluation, and management of the patient, the ability to continuously monitor and record tic events is the key to obtaining a patient-specific understanding of the disease. While reviewing and evaluating these monitoring data is a highly time- and cost-intensive process for doctors, the use of computer-assisted detection of tic movements can save time and cost, empower doctors to optimize and adjust medication responses, and help establish a good evaluation and management process for patients. Our work is the first application of a deep learning for video-based assessment of Tourette syndrome.

As the above experimental evaluations show, our video-based architecture possesses the ability to detect motor tic events in TS from videos acquired in a natural state. In the classification tasks, we detected two kinds of tics that occur most often in patients. Although the multiclass classification task involves limited motor tic categories in our dataset, it represents a unique result: to the best of our knowledge, no other similar research that has used surveillance video data for automatic tic recognition and classification exists. In the evaluation of subsequent results of the model, we defined some items that frequently appeared on the tic scales applied on these model outcomes and obtained consistent results with those from clinicians based on the MRVRS, which shows the ability to integrate observation-based scales or screening instruments for tics, although our current dataset limited a part of it. If we expand to audio data in the near future, it could be more comprehensive for developing an automatic rating scale of tics. For the binary classification task, it achieved good accuracy on video-clip-based recognition; however, it needs more video data for individual tests and other clinical data to support its outstanding performance in computer-aided diagnosis.

From our perspective, this work has application prospects from two main aspects: (a) automatic annotation of a video TS dataset. Because our classification task is based on small video clips, the task model can be used to prelabel the video and can then be checked by a doctor in a subsequent continuous data collection task, thereby reducing the doctor's labeling workload and accelerating the accumulation of labeled data. (b) Home-based health management applications: the extensive use of monitoring cameras makes it possible to extend this work to home-based health monitoring and management since acquiring video at home enhances the retrieval of objective tic expression [41]. In this case, object recognition and tracking, multiangle analysis, body tic detection, etc. all need to be considered and resolved. Furthermore, noise reduction and voice extraction are also significant for voice tic detection. A home-based tic surveillance system allows doctors and family members to better manage and provide more effective treatments for patients with tics who are undergoing long-term observation and treatment.

The inadequacy of labeled data is a clear limitation to future work and constitutes a weakness that we alleviate through unsupervised learning methods. We will continue to try to ameliorate this limitation by integrating the few-shot learning method, which has performed well on many tasks with only small amounts of available training data [42, 43]. Moreover, this work can be applied and expanded to multicenter data analysis similar to [44, 45]; a larger research platform may result in additional interesting research works.

## 5. Conclusions

In this work, we introduce the first application of a deep learning method that combines unsupervised and supervised learning for video-based facial tic motion detection in TS patients. The developed model achieved good classification results on both multiclass and binary classification tasks; it can both detect and classify facial tic behaviors. This study effectively utilized large amounts of unlabeled data, which greatly reduced the labeling workload. A subsequent quantification of tic behavior has potential clinical application value for early identification and auxiliary diagnosis and evaluation of treatment effects. In the future, more video data will be collected and used to evaluate our scheme.

## Figures and Tables

**Table 1:** Original TS video dataset.

Category	Labeled dataset	Unlabeled dataset
Videos	13	55
Minutes	136	709

**Table 2:** Evaluations of the multiclass classification task.

	Accuracy	Precision	Recall	F1-score
C3D [22]	0.7252 (±0.108)	0.7483 (±0.047)	0.7023 (±0.051)	0.7194 (±0.032)
TSN [39]	0.8988 (±0.117)	0.8354 (±0.089)	0.7284 (±0.054)	0.7600 (±0.070)
Ours-acc^1^	**0.9487 (±0.0298)** ^*∗∗*^	**0.8626 (±0.084)** ^*∗∗*^	**0.7878 (±0.106)** ^*∗*^	**0.7975 (±0.093)** ^*∗*^
Ours-f1^2^	0.9363 (±0.0390)	0.7628 (±0.209)	0.7362 (±0.198)	0.7391 (±0.198)

^1^Ours-acc means the proposed architecture with the watch-accuracy strategy. ^2^Ours-f1 means the proposed architecture with the watch-F1 strategy.  ^*∗*^*p* value <0.01;  ^*∗∗*^*p* value < 0.001.

**Table 3:** Evaluations of binary classification task.

	Accuracy	AUC_ROC	AUC_PR	Precision	Recall	F1-score
Ours-acc	0.8890 (±0.0458)	0.7532 (±0.080)	0.7035 (±0.138)	0.8057 (±0.103)	0.7532 (±0.103)	0.7634 (±0.093)
Ours-f1	**0.9057 (±0.0479)**	**0.7815 (±0.155)**	**0.7669 (±0.187)**	**0.8633 (±0.150)**	**0.7707 (±0.296)**	**0.7874 (±0.264)**

**Table 4:** Evaluations of some items of scales.

Test ID	Number of tic areas		Tic frequency (tics/min)		Time for evaluation (min)		
	Clinician	Our model	Clinician	Our model	Clinician	Our model	Clinician review
1	2	2	6	5	>40	<5	<5
2	2	2	6	7	>40	<5	<5
3	1	2	2	1	>30	<5	<5
4	1	1	40	37	>70	<5	<10
5	1	1	3	1	>30	<5	<5
6	1	1	9	12	>50	<5	<10
7	1	1	15	14	>60	<5	<5
8	1	1	0	0	>30	<5	<5
9	1	1	1	1	>30	<5	<5
10	1	1	11	8	>50	<5	<10
11	2	2	3	3	>30	<5	<5
12	1	0	0	0	>30	<5	<5
13	1	2	4	2	>40	<5	<5
*p * **value**	**0.7211**		**0.8666**		**<0.0001**		–

**Table 5:** Non-TS patient evaluation.

No.	Accuracy	Number of clips
1	0.9701	67
2	0.9531	192
3	0.9016	193
4	0.9890	91

## Data Availability

The TS video data used to support the findings of this study are restricted by the Ethics Committee of the Second Affiliated Hospital of Zhejiang University School of Medicine to protect the patient privacy. The data are not publicly available due to ethical restrictions.

## Supplementary Materials

Supplementary Materials Figure S1: Distribution diagram of the TS dataset, including the unlabeled dataset and the labeled dataset. The upper panel shows the frame distribution of every patient video, and the lower panel shows the time distribution of the videos in the TS dataset.
