# Supplementary figures and images for: Tic Detection in Tourette Syndrome Patients Based on Unsupervised Visual Feature Learning
Source: J Healthc Eng. 2021 Jun 7;2021:5531186. doi: 10.1155/2021/5531186 (PMC8203362; doi:10.1155/2021/5531186)

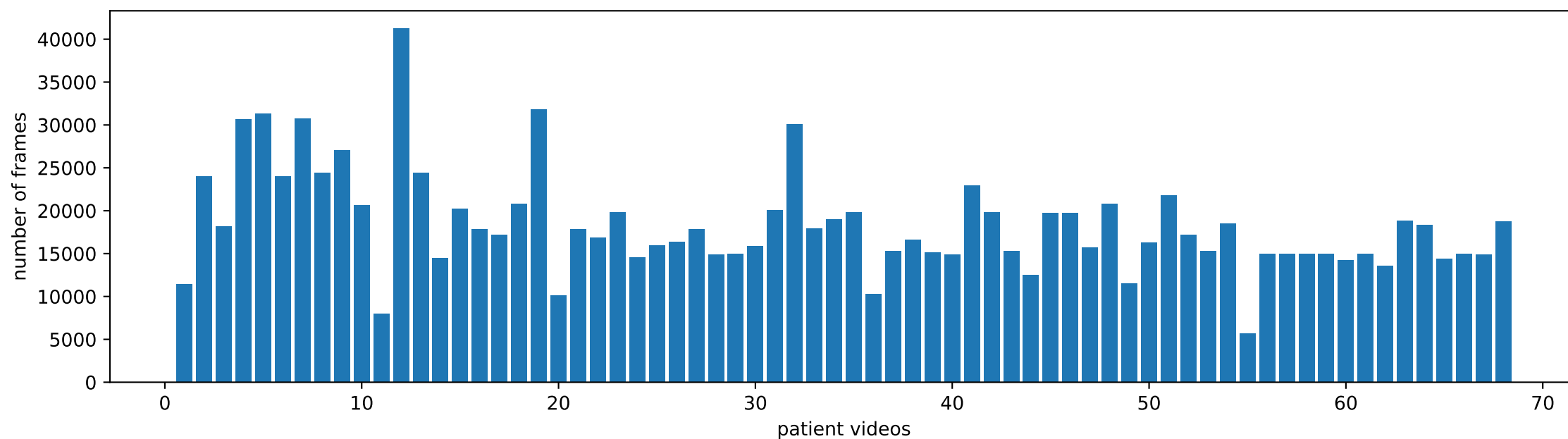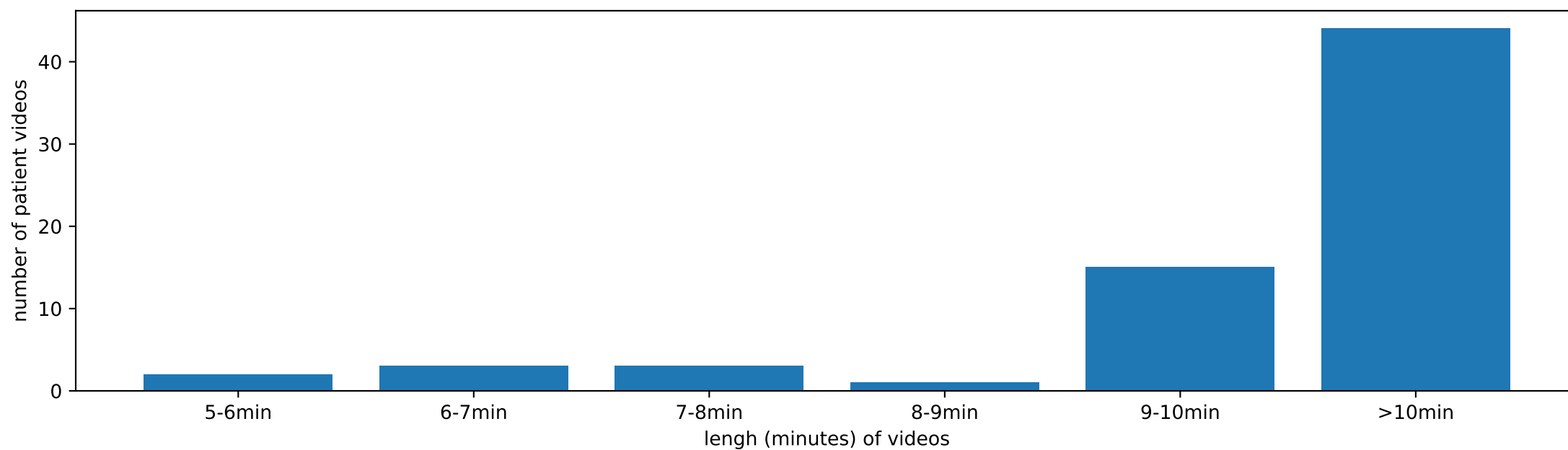

Supplement: Supplementary Materials — Figure S1: Distribution diagram of the TS dataset, including the unlabeled dataset and the labeled dataset. The upper panel shows the frame distribution of every patient video, and the lower panel shows the time distribution of the videos in the TS dataset. [file 5531186.f1.pdf]
